# Mental health and industry: Dynamics and perspectives

**DOI:** 10.4103/0972-6748.57850

**Published:** 2009

**Authors:** Kalpana Srivastava

**Affiliations:** Editor, IPJ

“Love and work are the cornerstones of our humanness.” Freud was perhaps one of the first to recognize the connection between work and mental health. Since his time (1856–1939), there has been research evidence of an important link between person’s mental wellbeing and productivity. Unfortunately, the worldwide trend of rise in mental ill health is alarming. Mental illness and addiction rank first and second in terms of causing disability in Canada, the United States and Western Europe when compared to all other diseases (e.g., cancer and heart disease). Mental health disability is the primary issue in 60–65% of disability insurance claims in Canada. (Stewart, W., Matousek, D., and Verdon, C. (2003). However, in India the insurance sector has not addressed this disability.

A healthy workplace leads to a good morale, and high motivation. There are linkages between mental health and other physical conditions. Some of them i.e. depression is linked to other physical and chronic conditions such as asthma, diabetes and hypertension. Employees experiencing job stress can triple the risk of disability associated with mental health illness, anxiety, substance use, back pain, injuries and infections.

In any organization, the workforce is its biggest asset. Without a mentally healthy workplace, the team will experience low morale, people will become cynical, stressed and anxious; physical health problems will increase, sickness levels will rise, productivity will drop and organizational climate will be affected. A mentally healthy workplace is one which is seen as a happy and friendly place to work. It has high productivity levels and is efficient, and is open to discussions about mental health issues.

The close investigation of the micro and macro level of organizational climate indicates that the process of illness begins at the micro level. Lewinsohn *et al*.,(1980) commented that the process from stressors to illness was interfered with by personal social skills and that social skills were important for mental health. In Japan, Takahashi (2003) suggested that social skills were important to perform actual health behaviors. Bandura (1982) also found that mental health status of employees is influenced by self-efficacy, self-management skills, and communication with superiors. Hence the role of individual factors cannot be overemphasized.

Proposed self-management skills by authors (Takahashi H, Kinoshita T, Masui S, Nakamura M, 1999) are considered the base of coping strategies because they include collecting information needed to carry out tasks, identifying core problems, and feasible pace and planning of such task. Irie *et al*., (1997) found that difficulty in dealing with stress and negative and malfunctional coping strategies were relative to the negative mental health of Japanese workers.

Bartel and Taubman(1979) were among the first to examine the relationship between mental health and labor market behavior. They explored the relationship between several diseases (including mental disorders) and individual earnings, wages, weekly hours worked, the probability of being out of the labor force, and the probability of being unemployed, using a sample drawn from a twins’ panel maintained by the National Academy of Science–National Research Council (NAS–NRC). Bartel and Taubman(1986) found that individuals diagnosed as either psychotic or neurotic had lower earnings, wages and weekly hours worked, and a greater probability of being out of the labor force. These results suggest that mental illness has a substantial impact in the labor market. Research questions in this area have examined the relationship between mental illness and labor market variables.

French MT, Zarkin GA (1998) carried out a study to explore the relationship between symptoms of emotional and psychological problems and employee absenteeism and earnings among employees at a large US worksite. The study revealed the role of the effects of emotional/psychological symptoms on two important labor market variables: absenteeism and earnings. Several specifications of the absenteeism and earnings equations were estimated to test the independent effect of emotional symptoms and the joint effects of emotional symptoms and other co-morbid conditions. The results suggest that employers should consider the productivity losses associated with workers’ mental health when designing worksite-based programs such as employee assistance programs (EAPs). Hence productivity is related with the mental health of the employees.

There is a negative relationship between absenteeism and mental health. In extreme cases, long-term stress or traumatic events at work may lead to psychological problems and be conductive to psychiatric disorders resulting in absence from work and preventing the worker from being able to work again. When under stress, people find it difficult to maintain a healthy balance between work and non-work life. The experience of work stress is a challenge to the health and safety of workers and to the healthiness of their organizations. The related terms which have been identified and studied are “Presenteeism”, presenteeism is defined as lost productivity that occurs when employees come to work but perform below par due to any kind of illness. While the costs associated with the absenteeism of employees have been long studied, the costs of presenteeism are newly being studied. The cost of absenteeism is obvious—100% of the worker’s productivity is lost each day the worker is not on the job; the cost of presenteeism is a more “hidden” cost because the worker is on the job but is not accomplishing as much (Lovell V 2004).

However, similar evidence in the Indian scenario is lacking. Some strategies are proposed to counter the ill effects of mental health. Some of the mental health intervention strategies are known as Mental Health First Aid techniques. “Mental Health First Aid’ are health enhancers based on solution-based strategies for coping with stress at the workplace. This was originally developed by the Centre for Mental Health Research at the Australian National University. Mental health awareness is the concept proposed by Mental Health First Aid techniques. This has been implemented in countries like England and Australia. The Mental Health First Aid refers to understanding how to preserve life where persons may be a danger to themselves or others and to provide help to prevent a mental health problem developing into a more serious state. Some of the objectives include raising awareness of mental health issues in the community and promoting the recovery of good mental health, providing comfort to a person experiencing a mental health problem and reducing stigma and discrimination.

Mental health first aid has been adapted and regulated by the National Institute for Mental Health in England (NIMHE) and England’s Care Services. There is a need to increase awareness in the area of mental health in the Indian scenario will help managers and executive cadre to identify signs of the mental ill health of employees and further help to intervene appropriately. Hence enhancement of awareness about mental health issues is essentially required for effective management and productive environment of the organization.
